# Infants’ Learning of Rule-Based Visual Sequences Predicts Language Outcome at 2 Years

**DOI:** 10.3389/fpsyg.2020.00281

**Published:** 2020-02-25

**Authors:** Roberta Bettoni, Valentina Riva, Chiara Cantiani, Massimo Molteni, Viola Macchi Cassia, Hermann Bulf

**Affiliations:** ^1^Department of Psychology, University of Milano-Bicocca, Milan, Italy; ^2^NeuroMI, Milan Center for Neuroscience, Milan, Italy; ^3^Child Psychopathology Unit, Scientific Institute, IRCCS E. Medea, Bosisio Parini, Lecco, Italy

**Keywords:** infants, language development, implicit learning, rule learning, syntax, lexicon

## Abstract

The ability to learn and generalize abstract rules from sensory input – i.e., Rule Learning (RL) – is seen as pivotal to language development, and specifically to the acquisition of the grammatical structure of language. Although many studies have shown that RL in infancy is operating across different perceptual domains, including vision, no studies have directly investigated the link between infants’ visual RL and later language acquisition. Here, we conducted a longitudinal study to investigate whether 7-month-olds’ ability to detect visual structural regularities predicts linguistic outcome at 2 years of age. At 7 months, infants were tested for their ability to extract and generalize ABB and ABA structures from sequences of visual shapes, and at 24 months their lexical and grammatical skills were assessed using the MacArthur-Bates CDI. Regression analyses showed that infants’ visual RL abilities selectively predicted early grammatical abilities, but not lexical abilities. These results may provide the first evidence that RL mechanisms are involved in language acquisition, and suggest that RL abilities may act as an early neurocognitive marker for language impairments.

## Introduction

Language is a fundamental and complex human ability containing several levels of structures, from the order of syllables within words to the order of words that build utterances. Newborn studies indicating postnatal retention of prenatal learning of mother’s speech sounds (e.g., DeCasper and Spence, 1986; May et al., 2011), as well as studies on few-month-old infants’ ability to discriminate and process the speech signal (e.g., Saffran et al., 1996; Marchetto and Bonatti, 2015) indicate that infants approach the complex task of language learning equipped with a set of neuropsychological and perceptual abilities that allow them to organize and give meaning to the linguistic input. These include domain-general cognitive mechanisms such as implicit learning, that is the ability to acquire structures from the environment to generate knowledge representations without intention to learn (Reber, 1967).

Implicit learning is not a unitary construct. Indeed, different kinds of learning mechanisms fall under the broad umbrella of implicit learning; statistical learning and rule learning are two examples. Statistical Learning (SL) refers to the ability to extract structural relations defined by statistical regularities from a continuous stream of input (Saffran et al., 1996); Rule Learning (RL) allows infants to detect abstract rules, and to generalize them to new exemplars that have no surface features in common with those on which learning took place (Marcus et al., 1999). These mechanisms are functional from the earliest stages of development (Gervain et al., 2008; Bulf et al., 2011), and are domain-general in nature, as they operate on both auditory – linguistic and non-linguistic (e.g., Marcus et al., 2007; Dawson and Gerken, 2009) – and visual input (e.g., Kirkham et al., 2002; Johnson et al., 2009).

Statistical learning and rule learning are both seen as pivotal for language development (Perruchet and Pacton, 2006; Romberg and Saffran, 2010; Aslin and Newport, 2014). Indeed, infants are exposed to speech streams in which there are not definitive acoustic cues for word boundaries or grammatical categories that help to detect words and syntax structures. Therefore, to gain lexical knowledge infants must parse speech streams by detecting highly predictable sequences of syllables that compose words, so as to associate their phonological forms to meaning. To gain syntactic knowledge, they must catch the relationship between words, and create an abstract representation of grammar categories – like subject, object or verb – so as to infer the system of rules that combines these unities of speech. Therefore, it has been hypothesized that the processing of statistical dependencies hidden within the linguistic input is critical to speech segmentation and vocabulary acquisition (Saffran et al., 1996; Perruchet and Pacton, 2006), whereas the ability to detect and represent abstract rules would mediate the extraction of the grammatical structure (Marcus et al., 1999; Peña et al., 2002; Endress and Bonatti, 2016). Accordingly, the association between early SL abilities and language acquisition has been demonstrated by longitudinal studies. For example, infants’ visual SL at 8 months of age predicts vocabulary comprehension at 13 months (Shafto et al., 2012), and a similar association was found between visual sequential learning at 6 months and receptive and productive vocabulary at 22 months (Ellis et al., 2014). While these studies indicate that infants’ SL abilities support the development of lexical skills, the impact of early RL abilities on grammatical acquisition remains to be explored.

Infants’ RL abilities were first investigated by Marcus et al. (1999) who presented 7-month-olds with a sequence of syllables that contained a repetition-based rule such as ABB (e.g., woffe), ABA (e.g., wofewo), or AAB (e.g., wowofe). After 2 min of exposure, infants were able to generalize the rule to novel syllables, showing that they had represented the rule-like pattern acquired during the learning phase. Under similar testing conditions, infants failed to learn abstract rules from non-speech sounds (musical tones, animal sounds, timbres), but succeeded in generalizing to non-speech sounds a rule they had previously extracted from speech sounds (Marcus et al., 2007). Similarly, 7-month-old infants succeeded in learning rules from non-speech tones when sequences were presented within a social context (i.e., a conversation between two human agents; Ferguson and Lew-Williams, 2016), and in the presence of inter-sensory redundancy delivered by social touch (i.e., touch sequences received from the experimenter; Lew-Williams et al., 2017). These findings indicate that RL in the auditory domain is enhanced in the presence of linguistic input or social signals.

Many studies have demonstrated that infants’ RL is fully operative in the visual domain as well, even in the absence of social cues. For example, 7-month-olds can extract and generalize abstract rules from visual sequences of familiar objects, such as images of animals (Saffran et al., 2007) and upright faces (Bulf et al., 2015), but also from visual sequences of unfamiliar geometrical shapes, at least when sequences are presented from left to right (Bulf et al., 2017). Overall, these pieces of evidence show that RL is a domain-general mechanism that operates across sensory modalities since early infancy.

Considering the availability of RL skills in early infancy and their potential role in language acquisition, we adopted a prospective design to investigate whether 7-month-olds’ visual RL abilities would predict infants’ grammatical skills at 24 months. Indeed, infants’ learning of ABB/AAB/ABA rule-like patterns from visual sequences is not altogether dissimilar from their learning of the grammatical structure of language, as both learning processes rely on the ability to keep track of the invariant positional relation of an item within a sequence, and to generalize this relation to novel elements (Mintz et al., 2002). For example, to learn an ABA rule-like structure, infants must notice that the first and the last elements of a 3-item sequence share the same surface features. In a similar way, infants extract the non-adjacent noun-verb-noun structure from sentences like “Lorenzo watches the computer,” a process that is crucial for the acquisition of syntactic structure categories (Gómez and Gerken, 2000). By focusing on the association between inter-individual variability in language acquisition and RL in the visual modality, we aimed to control for learning biases that may originate from infants’ perceptual expertise in processing linguistic sounds. Moreover, the use of visual stimuli allowed us to investigate the role of early domain-general learning abilities on later language acquisition (Karmiloff-Smith, 1998; Hollich et al., 2000).

The present study is a follow-up of an earlier study that investigated the role of spatial information on infants’ RL abilities (Bulf et al., 2017). In this earlier study, two groups of 7-month-olds were habituated to triplets of visual shapes that followed an ABB or an ABA rule and were presented sequentially on the screen. For one group, the shapes were presented sequentially from left to right, for the other group stimulus presentation occurred from right to left. Following the habituation phase, infants in both groups were presented with novel ABB and ABA triplets composed of new shapes. Results showed that infants learnt and generalized to the test sequences the rules presented during habituation when the sequences were presented from left to right, but failed with a right-to-left presentation. Indeed, only infants assigned to the left-to-right presentation condition showed a significant preference for the novel rule at test, while those assigned to the right-to-left condition showed no preference. This result replicates and extends previous evidence that spatial information affects infants’ ability to extract ordered information in increasing/decreasing triplets of numerical arrays: infants’ discriminated inversion in ordinal direction after habituation to left-to-right oriented numerical sequences but failed to do so when the sequences were right-to-left oriented (de Hevia et al., 2014). Overall, these findings show that a right-to-left orientation of visual sequences hinders infants’ serial order abilities, possibly as a result of infants’ exposure to early cultural practices that match the direction of the reading/writing system of their parents, which, in Western countries, is left-to-right oriented (see de Hevia et al., 2012 and de Hevia et al., 2014 for further discussion; see Göbel et al., 2018; McCrink et al., 2018 for evidence on the role of culture in shaping early spatial biases).

In the current study, we followed longitudinally the infants who were assigned to the left-to-right presentation condition of the Bulf et al. (2017) study, as this is the only experimental condition that triggered infants’ RL abilities. While Bulf et al. (2017) analyzed the performance of these infants at a group level, here we aimed to explore whether and how individual differences in early visual RL abilities are associated to later developing language skills at 24 months of age. We chose to assess language skills through parental reports, as they provide a continuous, comprehensive sampling of language abilities in ecological contexts and familiar situations, in which toddlers are more likely to talk (Dale, 1991). The Mean Length of Utterances (MLU) and the Number of Words Produced (Vocabulary) were collected as measures of, respectively, grammar and lexical skills. The relation between visual RL abilities and language skills was investigated through regression analyses. We expected that, if the ability to extract rule-like structures from visual input is involved in the extraction of the grammatical structure of language, infants’ novelty preference in the (left-to-right) visual RL task at 7 months would be selectively and positively associated with measures of utterances length. That is, we anticipated that infants who showed larger novelty preferences in the left-to-right RL task would score higher on MLU measures, in the absence of any relation with Vocabulary.

## Methods

### Participants

The sample included 24 of the original infants who participated in the left-to-right RL task of Bulf et al.’s (2017) study, for whom assessment of language skills was successful at 24 months, and six additional infants tested in the same experimental task used by Bulf et al. (2017). The final sample was thus composed of 30 infants (18 females) tested at 7 months (range = 7 months and 1 day- 8 months and 17 days) in the visual RL task, and re-evaluated at 24 months (range = 24 months and 3 days – 25 months and 8 days) for their expressive language skills by means of a parental questionnaire. The overall sample size was determined *a priori* using G^∗^Power (Faul et al., 2007). In order to obtain a medium effect size of 0.25 with α = 0.05 and power = 0.75, the sample size was estimated to be *N* = 30. All infants were full-term and monolingual, as their parents were both native Italian-speakers. For all infants, first-degree relatives had no certified diagnosis of specific language impairment. The research was conducted in accordance with Declaration of Helsinki, and the procedure was approved by the Ethical Committee of the University of Milano-Bicocca. All parents gave written informed consent for their infant’s participation.

#### Rule Learning Task

**FIGURE 1:**
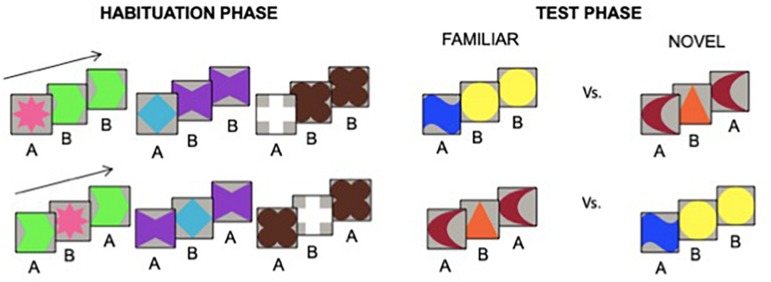
Examples of stimuli used in the habituation and test phases of the visual RL task performed by 7-month-old infants.

The stimuli and procedure used to test infants’ RL abilities are described in full in Bulf et al. (2017), Exp. 1; Figure 1. Infants were tested using an infant-controlled habituation procedure. Each habituation and test trial consisted of colored shapes organized into either ABB (adjacent late repetition of the B element) or ABA (non-adjacent repetition of the A element) triplets. Shapes within each triplet were presented sequentially on the screen from left to right with no accompanying sound, and each image disappeared before the onset of the next one (Figure 1). The experimenter recorded infant’s fixation by holding the mouse button whenever the infant fixated on the stimulus. Each trial continued until the infant looked continuously for a minimum of 500 ms and ended when the infant looked away for two consecutive seconds or looked for a maximum of 60 s. The habituation phase ended when the infant saw a maximum of 25 trials or met the habituation criterion, which was defined as a 50% decline in looking time on three consecutive trials, relative to the looking time on the first three trials. Half of the infants was randomly assigned to the ABB habituation condition, the other half to the ABA habituation condition. Following habituation, infants viewed six test trials in which triplets of novel shapes instantiating the ABA and ABB rules were presented alternately, each for three times, with half of the infants seeing the triplet instantiating the familiar rule first. Means of looking time (s) toward novel and familiar pattern were used as the dependent variable.

The image of the infant’s face was also recorded via a Mini-DV digital recorder; for about half of the infants (*N* = 13) looking times during test trials were coded offline by a second independent observer who was blind to the experimental condition. Inter-observer agreement (Pearson correlation) between the two observers who coded the data live or from digital recording, as computed on total fixation times on each of the six test trials, was *r* = 0.99, *p* < 0.001.

#### Language Measures

Infants’ language abilities were measured through the parent-administered questionnaire ‘Primo Vocabolario del Bambino (PVB), Parole e Frasi’ (Caselli and Casadio, 1995), which is the Italian version of the ‘MacArthur-Bates Communicative Development Inventories (CDI): Word and Sentences’ (Fenson et al., 2006). The CDI is one of the most commonly used assessment tools in the study of early language development, with high concurrent and predictive validity (Law and Roy, 2008). The PVB questionnaire provides measures of both lexical abilities (i.e., Expressive Vocabulary, EV) and grammar skills (i.e., MLU) in toddlers aged 16–30 months.

##### Expressive vocabulary (EV)

Early lexical abilities are typically inferred from the amplitude of the child’s EV. The PVB quantifies EV as the number of words marked by parents in a list of 670 words as being actually produced by the child, with higher EV scores indicating better expressive vocabulary abilities. Toddlers with delays in EV (i.e., late talkers) often show language and reading difficulties at school (Rescorla and Schwartz, 1990; Rescorla, 2011; Hawa and Spanoudis, 2014). Moreover, early EV scores are widely considered as a reliable early marker of language difficulties and neurodevelopmental disorders (Rice et al., 2005; Paul and Roth, 2011).

##### Mean length of utterance (MLU)

Early grammar abilities are typically inferred from the length of the child’s utterances (i.e., MLU). The PVB quantifies MLU as the mean number of words included in three utterances provided by the parents as examples of the longest utterances that the child produces score, with higher MLU scores indicating better early grammatical abilities. MLU is widely used as a benchmark of language acquisition, and, when measured before the age of 3 years, it predicts later grammar abilities (Devescovi and Pizzuto, 1995). MLU is also considered a reliable marker to identify children with language impairments (i.e., Rice et al., 2010; Rudolph and Leonard, 2016).

### Data Analysis

To investigate infants’ performance in the left-to-right RL task, we analyzed the looking times recorded during the habituation and the test trials of the task. To compare infants’ performance during habituation to ABB and ABA sequences, mean habituation looking times were entered into a 2-way ANOVA with habituation rule (ABB vs. ABA) as the between-participants factor and habituation trials (first vs. last three) as the within-participants factor. To determine whether in test infants were able to discriminate the familiar from the novel rule-like patterns, total test looking times were entered into a 4-way ANOVA with habituation rule (ABB vs. ABA) and test order (familiar first vs. novel first) as between-participants factors, and test trial pair (first vs. second vs. third) and test trial type (novel vs. familiar) as within-participants factors. To investigate the predictive role of early visual RL skills on language abilities at 24 months of age, we conducted a path analysis including infants’ performance at discriminating between the novel and familiar rule-like patterns (i.e., Rule Discrimination score) and their EV and MLU raw scores. Rule Discrimination scores were obtained for each infant by computing the difference between total looking times on the novel test trials and total looking times on the familiar test trials. Path analyses are commonly used to examine the relationship among variables and test theoretical causal models when multiple variables are involved. Accordingly, we included Rule Discrimination scores from the visual RL task performed at 7 months as the independent variable, and EV and MLU scores obtained from the same infants at 24 months as the dependent variables.

## Results

### Visual Rule Learning Abilities at 7 Months

All infants reached the habituation criterion with a mean of 8.30 trials (*SE* = 0.54) and a mean looking time of 96.94 s (s) (*SE* = 8.52). The 2 (habituation rule: ABB vs. ABA) × 2 (Habituation trials: first three vs. last three) ANOVA on mean habituation looking times revealed an overall significant decline in infants’ mean looking times from the first three (*M* = 17.37 s, *SE* = 1.48) to the last three habituation trials (*M* = 6.79 s, *SE* = 0.65), *F*(1,28) = 109.68, *p* < 0.001, η^2^ = 0.797. Habituation times were not affected by the rule (ABB or ABA) delivered during the habituation phase (all *p_*s*_* > 0.2).

The 4-way ANOVA on looking times at test, with habituation rule (ABB vs. ABA) and test order (familiar first vs. novel first) as between-participants factors, and test trial pair (first vs. second vs. third) and test trial type (novel vs. familiar) as within-participants factors, revealed a main effect of test trial type, *F*(1,26) = 12.78, *p* = 0.001, η^2^ = 0.330, as infants looked overall longer to the novel test sequences (*M* = 9.88 s, *SE* = 1.10) than to the familiar ones (*M* = 7.10 s, *SE* = 0.85). There was also a main effect of test trial pair, *F*(2,52) = 11.61, *p* < 0.001, η^2^ = 0.309, revealing a decrement in infants’ overall looking times from the first (*M* = 10.80 s, *SE* = 1.32) to both the second (*M* = 8.02 s, *SE* = 0.99; *p* = 0.014) and the third (*M* = 6.64 s, *SE* = 0.69; *p* < 0.001) trial pairs. No other effects or interactions were significant (all *p_*s*_* > 0.1).

These results indicate that infants were capable to detect and represent the rule-like patterns instantiated by the habituation sequences, and to generalize them to the novel shapes at test.

### Language Abilities at 24 Months

Measures of early grammar abilities were obtained through the PVB questionnaire at 24 months of age for all the 30 infants who previously took part in the visual RL task, whereas measures of lexical abilities were obtained for only 29 infants, because one parent failed to complete the relevant section of the questionnaire.

#### Expressive Vocabulary (EV)

Expressive vocabulary was assessed through the number of words produced (EV, *M* = 270.10, *SE* = 28.01, range = 24–640; Skewness = 0.269; Kurtosis = −0.483). The distribution of the obtained EV scores is representative of the distribution of language abilities at 24 months in the general population, which includes 8% of late talkers, as defined by a vocabulary size less than 50 words (*N* = 2 out of 29, corresponding to 6.90%).

#### Mean Length of Utterance (MLU)

Mean length of utterance was assessed through the mean length of the spontaneously produced utterances (MLU, *M* = 3.75, *SE* = 0.46, range = 0.0–8.67; Skewness = −0.019; Kurtosis = −0.542). The obtained MLU scores matched the normative data available for the Italian population (Caselli and Casadio, 1995).

### Relation Between Visual Rule Learning and Language Abilities

**FIGURE 2:**
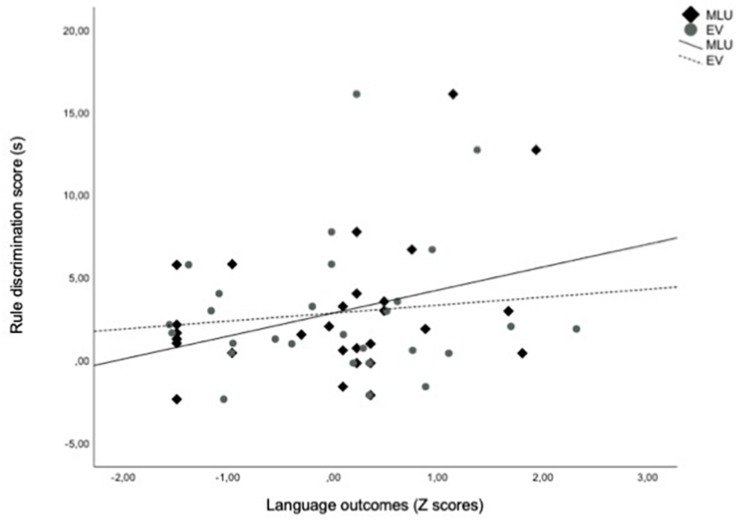
Infants’ Rule Discrimination scores (total looking times on the novel test trials minus total looking times on the familiar test trials) recorded at 7 months plotted as a function of *z*-transformed MLU (Mean Length of Utterances) and EV (Expressive Vocabulary) scores measured at 24 months.

We observed a significant positive correlation between EV and MLU scores at 24 months of age, *r*(30) = 0.754; *p* < 0.001. To evaluate the predictive association between infants’ performance in the visual RL task and language outcome at 24 months, we conducted a path analysis considering infants’ Rule Discrimination score in the RL task as the independent variable upon MLU and EV, which were both entered as dependent variables. The path analysis was performed using structural equation modeling (SEM) as implemented in the M-PLUS software version 7 (Mutheìn and Mutheìn, 2014). SEM is used to examine relationships among variables and test theoretical causal models when multiple variables are involved. All the relationships among variables in the model are tested together and all of the paths can be compared with each other in terms of the degree of importance of each variable (Pedhazur and Kerlinger, 1982). We used the method of maximum likelihood that tolerates departures from normality, especially if skewness values are below |2| and kurtosis values are below |7| (West et al., 1995). The model provided a good fit to the data (*X*^2^(3) = 4.72, *p* = 0.094; RMSEA = 0.000, CI (90%) = 0.000 − 0.000; CIF = 1.00, SRMR = 0.000), and explained 15% of the variance of the MLU outcomes, thus accounting for a link between early visual RL abilities and later developing grammatical skills (Figure 2). No outliers were detected according to Cook’s distance (Cook’s *D* < 1; Cook and Weisberg, 1982).

**FIGURE 3:**
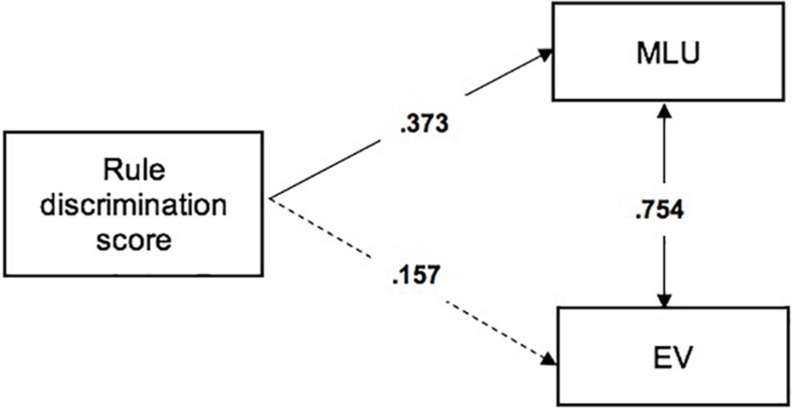
Regression model showing that Rule Discrimination scores at 7 months predicts MLU (Mean Length of Utterances) scores, but not EV (Expressive Vocabulary) scores at 24 months.

Standardized estimates of path coefficients are depicted in Figure 3. The model showed a significant path coefficient from Rule Discrimination score in the RL task to MLU (Beta = 0.373; *p* = 0.036). In contrast, the Rule Discrimination score in the RL task did not predict EV outcomes (Beta = 0.157; *p* = 0.379), indicating that early RL abilities specifically predicted early grammatical aspects of language development. In particular, higher novelty preference scores at 7 months of age predicted better early grammatical skills at 24 months of age.

## Discussion

It is widely accepted that implicit learning plays a key role in language development (Conway and Pisoni, 2008; Arciuli and Torkildsen, 2012). Although the power of implicit learning in explaining a wide range of linguistic functions is gaining increasing support (e.g., Saffran and Kirkham, 2018), relatively little research has attempted to link individual differences in language acquisition to individual differences in implicit learning skills by adopting a longitudinal perspective. Two recent studies have shown that visual SL abilities measured at 6–8 months predict later vocabulary at 13–22 months (Shafto et al., 2012; Ellis et al., 2014). However, evidence of predictive associations between early RL abilities and inter-individual variability in language comprehension and/or production skills is missing. Here, we show a relationship between preverbal infants’ ability to extract and generalize visual structural regularities and later developing grammatical skills: infants who were better at learning visual rule-based sequences at 7 months of age received higher MLU scores by the end of the second year of life. Specifically, the regression model revealed that infants’ RL abilities explained 15% of the variance of the MLU outcome, but did not predict the EV outcome, even though MLU and EV scores were highly correlated. Therefore, the present finding suggests that visual RL abilities are specifically related to the development of early grammatical skills, while they do not predict lexical skills as measured by vocabulary size.

The positive correlation between MLU and EV scores in our data confirms the well-established relation between grammatical competences and vocabulary size (Caselli et al., 1999; Tomasello, 2009). Nevertheless, the finding of a selective relation between RL abilities and early grammatical skills suggests that the development of grammar could be at least partially driven by general learning processes, and not entirely depend upon the acquisition of lexical knowledge (Skeide and Friederici, 2016). Indeed, there is evidence that many months before infants start producing their first words, they are sensitive to some aspects of grammar. For example, infants between 2 and 8 months are able to discriminate two sentences based on differences in word order (Mandel et al., 1996), and 6-month-olds can distinguish between nouns and verb phrases when prosodic cues are available (Soderstrom et al., 2003). There is also neuroimaging evidence for similar patterns of activation in 3-month-old infants and adults during a sentences repetition task involving superior temporal and inferior frontal regions, which are part of Broca’s area (Dehaene-Lambertz et al., 2006). Broca’s area is involved in the analysis of grammatical and semantic information (Hickok and Poeppel, 2007), and its activation in 3-month-old infants suggests that a sentence learning mechanism is already at work even before the onset of the babbling stage. Our findings suggest that RL might be one of the main general learning mechanisms supporting the development of grammar skills, as it allows infants to construct abstract (Kabdebon and Dehaene-Lambertz, 2019) and hierarchical (Kovács and Endress, 2014) representations from sequential streams of items, and to generalize these representations to novel items and contexts (Rabagliati et al., 2019). These abilities resemble very closely those needed to face the learning challenge posed by the extraction of grammatical structures from the linguistic input.

One limit of the current study is that language skills at 24 months were assessed solely through parental reports. Although a direct assessment of language development may have been a valid alternative, parental reports are particularly suitable to be used with toddlers in the early stages of language production, when the familiarity and diversity of everyday situations are critical in obtaining a large enough language sample (e.g., Sachse and Von Suchodoletz, 2008). Moreover, the PVB questionnaire has high concurrent and predictive validity, as it discriminates toddlers with late language emergence vs. toddlers with typical language development, and infants at high-risk vs. low-risk for language impairments (Law and Roy, 2008). It has been shown that parents’ reports of toddler’s EV are highly correlated with concurrent and later measures of language development (Feldman et al., 2005). In addition, MLU scores accurately predict grammatical abilities, specifically when the scores are obtained before the age of 3 years (Devescovi and Pizzuto, 1995). Future studies should replicate and extend the present findings by assessing language comprehension and production at later ages (e.g., 36 months), when vocabulary spurt has occurred and complex grammatical skills are emerging. This would allow to understand whether RL continues to support the acquisition of grammar when children’s syntax understanding and production is applied to more complex hierarchical structures.

The present study contributes to our understanding of the mechanisms that subserve language learning as it points to the idea that infants are equipped with a set of domain-general learning abilities that play a critical role in boosting language development (Karmiloff-Smith, 1998; Hollich et al., 2000). In turn, this suggests that the acquisition of language skills does not rely on a language-specific device, in line with the available evidence of the presence of a tight link between language acquisition and early domain-general abilities, such as memory (Colombo et al., 2004), attention (Rose et al., 2009), and auditory processing (Cantiani et al., 2016). It is worth noting that, unlike previous studies that took into consideration a single measure of language (i.e., vocabulary; Shafto et al., 2012; Ellis et al., 2014), we measured the relation between visual RL and two different components of language, i.e., vocabulary and early grammar. Given the multi-component nature of language, measuring different aspects of language development within the same study is critical to the understanding of how different implicit learning mechanisms in the earliest stages of development may contribute to language acquisition.

Another important aspect of the current results is the implications they have for the understanding of atypical trajectories of language development. Indeed, the sensitivity of the visual RL task to discriminate individual differences in language acquisition might candidate this task as a screening tool for the identification of infants at high-risk for language impairments. While previous research showed a deficit in statistical learning of speech streams in children with language impairments (Evans et al., 2009) and in infants at risk for dyslexia (Kerkhoff et al., 2013), the current study is the first to suggest that the assessment of infants’ RL in the visual modality could act as a marker task for language disabilities. Future studies shall investigate further this possibility, by disentangling whether language impairments are specifically related to difficulties in the processing of the linguistic input *per se*, or, as the current results suggest, are at least partially linked to domain-general learning difficulties.

## Data Availability Statement

The datasets generated for this study are available on request to the corresponding author.

## Ethics Statement

The studies involving human participants were reviewed and approved by the Committee for Research Evaluation, Department of Psychology, University of Milano-Bicocca. Written informed consent to participate in this study was provided by the participants’ legal guardian/next of kin.

## Author Contributions

RB conceived and designed the study, collected and analyzed the data, and drafted the manuscript. VR contributed to the data analysis and interpretation, and to the writing of the manuscript. CC supervised the data collection and contributed to the study design, data interpretation, and the writing of the manuscript. MM contributed to the planning of the study design. VM contributed to data interpretation and the writing of the manuscript. HB conceived the study, contributed to the study design, supervised the data collection, and contributed to the writing of the manuscript.

## Conflict of Interest

The authors declare that the research was conducted in the absence of any commercial or financial relationships that could be construed as a potential conflict of interest.
